# Description of *Mesopolobusaskewi* sp. nov. (Hymenoptera, Pteromalidae), with notes on the fauna of Asaphesinae and Pteromalidae (Hymenoptera, Chalcidoidea) collected from foliage of *Piceaabies* (L.) H. Karst. in Bulgaria

**DOI:** 10.3897/BDJ.12.e139403

**Published:** 2024-12-03

**Authors:** Ivaylo Todorov, Mircea-Dan Mitroiu, Aneliya Bobeva, Peter Boyadzhiev

**Affiliations:** 1 Institute of Biodiversity and Ecosystem Research, Bulgarian Academy of Sciences, Sofia, Bulgaria Institute of Biodiversity and Ecosystem Research, Bulgarian Academy of Sciences Sofia Bulgaria; 2 Alexandru Ioan Cuza University, Faculty of Biology, Iasi, Romania Alexandru Ioan Cuza University, Faculty of Biology Iasi Romania; 3 Department of Zoology, University of Plovdiv, Plovdiv, Bulgaria Department of Zoology, University of Plovdiv Plovdiv Bulgaria

**Keywords:** Chalcidoidea, new species, new records, Bulgaria, mountain fauna, Norway spruce

## Abstract

**Background:**

*Mesopolobus* Westwood, 1833 consists of about 135 valid species worldwide. After the fundamental monograph of Graham (1969), 12 species have been described from continental Europe and three species have been described from the Canary Islands and Malta. Amongst them, one species, *Mesopolobusblascoi* Askew, 1994, has been synonymised under *Mesopolobusmaculipennis* (Mercet, 1923). Only eight species have been reported from Bulgaria to date.

**New information:**

Here we describe a new species, *Mesopolobusaskewi* sp. nov. and present new data on the Bulgarian chalcidoid fauna obtained by sampling in foliage of the Norway spruce, *P.abies*. *Mesopolobusaskewi* sp. nov. can be distinguished from the most morphologically similar species, *M.longicollis* Graham, by the following characters: clypeus with deeper emargination, fore wings with basal vein having complete row of setae, head blue to bluish-green, mesosoma bluish-green to green with coppery reflections, legs after coxae mostly fulvous, only distal one-fifth of meso- and metatibiae yellowish, protarsi with fifth segment yellowish, only tarsal claws fuscous, venation pale testaceous. Furthermore, we identified nine valid species of the family Pteromalidae belonging to four genera – *Mesopolobus* (three spp.), *Pachyneuron* (one sp.), *Stenomalina* (one sp.) and *Trichomalus* (four spp.) and one species of subfamily Asaphesinae (Chalcidoidea, *incertae sedis*), all represented in our samples by many specimens and none having previously been reported as associated with foliage of the Norway spruce. Three of the species are new records for the Bulgarian fauna.

## Introduction

Norway spruce, *Piceaabies* (L.) H. Karst. (Pinaceae), is a Palearctic coniferous tree species distributed between the Alps in France and the foothills of the Urals. Its typical forests grow from 69°47’N latitude in Norway in the north to 41°45’N in Macedonia and Greece in the south, mostly in mountainous areas in central and south-eastern Europe, but also at lower altitudes in the Scandinavian Peninsula (Boratyńska 2007). In Bulgaria, the stands of *P.abies* cover about 158,000 ha and are amongst the most important forests in the mountain regions of the western part of the country (Panayotov et al. 2015).

Having high economic and ecosystem value in forestry, *P.abies* has been in the focus of many studies and the insect fauna associated with spruce in Europe has been reported by many authors in the past. However, these investigations have dealt mostly with conophagous and seed species, as well as on saproxylic and xylophagous beetles and their natural enemies (Balazy 1968, Yunap 1986, Weslien 1992, Kozioł 2000, Seifert et al. 2000, Lakatos and Thuróczy 2002, Lozan and Zeleny 2003, Hilszczański et al. 2005, Hedgren 2007, Kozioł 2010, Lotfalizadeh 2012, Rosenberg et al. 2015, Vanická et al. 2020 etc.) with little attention to the parasitoid communities inhabiting the tree crown.

Pteromalidae are known to form a considerable part of the hymenopterous parasitoid complex of spruce pests. To date 23 species (Noyes 2019, Wiśniowski and Jirak-Leszczyńska 2021) have been reported from *P.abies* in various European countries (Ohnesorge 1962, Bakke 1963, Pettersen 1976, Askew 1980, Grodzki 1997, Witteczek 2000, Pettersson 2001, Lakatos and Thuróczy 2002, Hoffmann and Schmutterer 2003, Grodzki 2009, Milosavljević et al. 2021). In Bulgaria, pteromalid fauna associated with spruce has not been intensively studied and only four species have been recorded from wood samples, mostly during investigations on bark beetles for the purpose of forestry (Georgiev and Takov 2005, Georgiev and Stojanova 2006).

*Mesopolobus* Westwood, 1833 is one of the largest genera amongst all Pteromalidae and consists of about 135 valid species worldwide (Noyes 2019, Nieves-Aldrey et al. 2020, László et al. 2021). It was firstly established by Westwood (1833), based on *M.fasciiventris*, by monotypy. After the publication series of von Rosen (von Rosen 1958a, von Rosen 1958b, von Rosen 1959a, von Rosen 1959b, von Rosen 1960, von Rosen 1961a, von Rosen 1961b, von Rosen 1962, von Rosen 1966) and the fundamental monograph of Graham (1969), 12 species have been described from continental Europe (Bouček 1974, Szelényi 1982, Askew 1994, Gijswijt 1994, Garrido and Nieves Aldrey 1996, Askew and Blasco-Zumeta 1997, Askew and Lampe 1998, Baur et al. 2007, Nieminen et al. 2018, Nieves-Aldrey et al. 2020, László et al. 2021) and three species have been described from the Canary Islands and Malta (Gijswijt 1990, Báez and Askew 1999, Dorchin et al. 2014). Amongst them, one species, *Mesopolobusblascoi* Askew, 1994, has been synonymised under *Mesopolobusmaculipennis* (Mercet, 1923) (Garrido and Nieves Aldrey 1996).

In the present paper, we describe a new distinctive species of genus *Mesopolobus*, namely *Mesopolobusaskewi* and contribute to the knowledge of the pteromalid fauna associated with *P.abies* from Bulgaria with 10 new parasitoid-plant associations. The new species is discussed and compared to the most similar ones described in *Mesopolobus* after Graham’s revision (Graham 1969). Brief data about the known biology and distribution of the rest of the species are also presented.

## Materials and methods

### Collection of insects and species determination

All samples were collected by sweep netting from the lowest branches of the trees in forest stands of *P.abies* during July and August in the years 2019, 2020 and 2021. Selected localities were situated between 1014 and 2155 m above sea level in the Western Rodope Mountains and Stara Planina Mountains (= Balkan Mountains), Bulgaria. Gathered chalcidoids were caught individually by aspirator and fixed in 96% ethanol. The rest of the non-target insects in the net bag were released alive back in the field. The materials were dehydrated using absolute ethanol (Merck KGaA, CAS-No: 64-17-5, Germany) in the laboratory, treated with hexamethyldisilazane (Sigma-Aldrich®, USA, 99.9%) for two soaks of 1/2 hour each, air dried and mounted on card points for further observation. The specimens were studied under a Carl Zeiss Discovery.V8 stereomicroscope at 25–80× and photographed using a digital camera Canon EOS 200D attached to the intermediate photo tube by a T2-T2 DSLR 1.6 adapter. Stacking process was generated through Helicon Focus (v.7.5.8 Pro). Specimens used for scanning electron microscopy were mounted on brass stubs and coated with gold. SEM-micrographs were taken with a Lyra I XMU (Tescan) scanning electron microscope (with Quantax 200 Bruker detector) at 10 kV.

Genus and species identification followed the keys in Delucchi and Graham (1956), Graham (1969), Askew (1980) and Bouček and Rasplus (1991). We used Noyes (2019), Burks et al. (2022) and UCD Community (2023) for nomenclature verification and data source concerning species distribution. Terminology of body parts, sclerites and integumental surface sculpturing followed Eady (1968), Graham (1969), Harris (1979), Wibel et al. (1984) and Gibson et al. (1997). Description of the new species was prepared following, to some extent, the manner of Graham (1969), Dorchin et al. (2014) and Nieves-Aldrey et al. 2020. All discussions concerning differences between *M.askewi* sp. nov. and species described after Graham (1969) are based on the original author’s descriptions.

### Molecular analysis

Total DNA was isolated from an individual insect using the DNeasy Blood & Tissue Kit following the manufacturer’s instructions. The extracted genomic DNA served as a template for amplifying two mitochondrial markers: Cytochrome c oxidase subunit I (COI) and Cytochrome b (CYTB). The primers used for polymerase chain reactions (PCR) were as follows: for COI – forward pF2 5’-ACCIGTDATRATRGGDGGITTYGGDAA-3’ and reverse 2413d 5’-GCTADYCAICTAAAAATYTTRATWCCDGT-3’ (Lohse et al. 2010) and for CYTB – forward CP1 5’-GATGATGAAATTTTGGATC-3’ and reverse CB2 5’-ATTACACCTCCTAATTTATTAGGAAT-3’ (Cruaud et al. 2010). Both reactions were performed using HotStarTaq Plus Master Mix (Qiagen, Inc.), following a temperature profile that included: initial denaturation step at 95°C for 5 minutes, followed by 5 cycles of 95°C for 1.5 minutes, 44°C for 1 minute and 72°C for 1.5 minutes and then 30 cycles of 95°C for 1.5 minutes, 48°C for 1 minute and 72°C for 1.5 minutes with a final extension at 72°C for 5 minutes. The presence of amplicons was confirmed using 2% agarose gel stained with GelRed (Biotium Inc.), with 3 μl of the reaction mixture loaded. The amplified fragments were sequenced by Macrogen Ltd. (Amsterdam, the Netherlands) from both the 5’ and 3’ ends. The obtained sequences were edited, assembled and aligned using CodonCode Aligner version 8.0.2 (CodonCode, Dedham, MA, USA). Finally, the processed sequences were compared to the available ones in the GenBank database.

### Additional material examined

Five specimens of *Mesopolobuslongicollis* Graham, 1969 - 3 females and 2 males, were provided for us by Dr. Richard Askew for comparison and are currently deposited in the entomological collection of the Institute of Biodiversity and Ecosystem Research. They are all labelled as follows: “England: Ainsdale, Lancashire; collected in 1977; leg Sulaiman Hanapi; em. V. 1978 from gall of *Iteomyiamajor*”.

### Deposition of specimens

Type specimens of *Mesopolobusaskewi* sp. nov. and most of the specimens belonging to other species obtained in this study are deposited in the entomological collection of the Institute of Biodiversity and Ecosystem Research (Bulgarian Academy of Sciences, Sofia, Bulgaria). Ten specimens of *M.askewi* sp. nov. are deposited in the personal collection of R. R. Askew. Less numerous materials are deposited in MICO (Mitroiu Collection) in the Faculty of Biology, Alexandru Ioan Cuza University (Iasi, Romania).

## Taxon treatments

### Mesopolobus askewi

Todorov sp. nov.

B4F99402-5129-5CF8-810C-2CC35FF57A2F

03A61CA1-AFCF-49E5-9CA2-F2DE94DAAEA3

#### Materials

- **Type status:** Holotype. **Occurrence:** recordedBy: Ivaylo Todorov leg.; sex: female; occurrenceID: DEF0892C-3067-52BB-8ACD-B3D528106BA5; **Taxon:** scientificName: Mesopolobusaskewi; **Location:** country: Bulgaria; locality: Western Rhodope Mts, near Pamporovo; verbatimElevation: 1580 m; locationRemarks: coniferous forest; decimalLatitude: 41.62250; decimalLongitude: 24.70100; **Event:** eventDate: 23.VIII.2018
- **Type status:** Paratype. **Occurrence:** recordedBy: Ivaylo Todorov leg.; sex: 4 females; preparations: whole specimens, mounted on card points; occurrenceID: 96752C8D-DA46-5DEE-BB45-62EB4C77E46F; **Taxon:** scientificName: Mesopolobusaskewi; **Location:** country: Bulgaria; locality: Western Rhodope Mts, near Pamporovo; verbatimElevation: 1580 m; locationRemarks: coniferous forest; decimalLatitude: 41.62250; decimalLongitude: 24.70100; **Event:** eventDate: 23.VIII.2018
- **Type status:** Paratype. **Occurrence:** recordedBy: Ivaylo Todorov leg.; sex: 1 female; preparations: dissected for SEM observation; occurrenceID: 3FBD0FDC-4838-56E2-AC29-B1A1BF174D41; **Taxon:** scientificName: Mesopolobusaskewi; **Location:** country: Bulgaria; locality: Western Rhodope Mts, near Pamporovo; verbatimElevation: 1580 m; locationRemarks: coniferous forest; decimalLatitude: 41.62250; decimalLongitude: 24.70100; **Event:** eventDate: 23.VIII.2018
- **Type status:** Other material. **Occurrence:** recordedBy: Ivaylo Todorov leg.; sex: 4 females; occurrenceID: 1E4CECB3-0046-590B-92AC-30D06C8F118D; **Taxon:** scientificName: Mesopolobusaskewi; **Location:** country: Bulgaria; locality: Western Rhodope Mts, near Pamporovo; verbatimElevation: 1580 m; locationRemarks: coniferous forest; decimalLatitude: 41.62250; decimalLongitude: 24.70100; **Event:** eventDate: 23.VIII.2018
- **Type status:** Other material. **Occurrence:** recordedBy: Ivaylo Todorov leg.; sex: 3 females; occurrenceID: 71B4F34A-C08E-59EB-98CE-AC9F046897E1; **Taxon:** scientificName: Mesopolobusaskewi; **Location:** country: Bulgaria; locality: Western Rhodope Mts, near Perelik hut; verbatimElevation: 2000 m; locationRemarks: coniferous forest; decimalLatitude: 41.60518; decimalLongitude: 24.59265; **Event:** eventDate: 20.VIII.2021
- **Type status:** Other material. **Occurrence:** recordedBy: Ivaylo Todorov leg.; sex: 5 females; occurrenceID: 475AD205-C6E7-559E-A34C-68C080FB62BE; **Taxon:** scientificName: Mesopolobusaskewi; **Location:** country: Bulgaria; locality: Stara Planina: Shipchenska Mts, near Uzana hut; verbatimElevation: 1243 m; locationRemarks: coniferous forest; decimalLatitude: 42.75800; decimalLongitude: 25.23427; **Event:** eventDate: 08.VII.2020
- **Type status:** Other material. **Occurrence:** recordedBy: Ivaylo Todorov leg.; sex: 1 female; occurrenceID: D878612E-EB2A-5481-8761-39CD48AF17E5; **Taxon:** scientificName: Mesopolobusaskewi; **Location:** country: Bulgaria; locality: Stara Planina: Troyanska Mts, near Beklemeto; verbatimElevation: 1433 m; locationRemarks: coniferous forest; decimalLatitude: 42.78280; decimalLongitude: 24.62160; **Event:** eventDate: 17.VIII.2021

#### Description

Length: 1.7–2.1 mm.

Colouration (Fig. 1a). Head blue to bluish-green. Mesosoma bluish-green dorsally, pro-, meso- and metapleuron, as well the lateral panels of pronotum, green with coppery reflections; propodeum bluish-green, shiny; gaster brownish, with bluish-green metallic tints dorsally. Antenna with scape, pedicel and anelli fulvous, funicular segments yellowish beneath, dorsally pale testaceous with weakly infuscate proximal half. Mandibles and palps pale yellow, mandibular teeth brown. Coxae dorsally coloured as mesosomal pleura, ventral surface light brownish. Legs with femora and protibiae fulvous, meso- and metatibiae mostly fulvous with distal one-fifth yellowish; protasi fully yellowish with only tarsal claws fuscous, meso- and metatarsal segments 1-4 yellowish, 5^th^ segments fuscous. Wings clear, venation pale testaceous.

Tegument. Head with upper face and vertex reticulate; lower face and genae reticulate to strigulate-reticulate; clypeus striate, striation not extending beyond sclerite margins; supraclypeal area reticulate; antennal scape and pedicel imbricate; Mesosoma: Pronotal collar and mesonotum reticulate, metanotum with lateral panels and dorsellum smooth and shiny; propodeum with median area between plicae weakly sculptured, shiny, median carina complete; propodeal plicae complete, sharp anteriorly and posteriorly, more rounded in the anterior third (Fig. 2f); nuchal strip short and smooth; upper mesepimeron faintly, but visibly reticulate (Fig. 1e), lower mesepimeron and mesepisternum moderately finely reticulate; Gaster: Tergite 1 smooth and shiny, second and third tergites in proximal 1/4^th^, fourth tergite in proximal half – with very shallow alutaceous sculpture, tergites 5-7 finely alutaceous on entire surface; all sternites finely alutaceous.

Head. 1.11-1.16x breadth of mesosoma, in dorsal view 1.94-2.0x as broad as long, temples 0.24-0.27x the length of eye (Fig. 1b), head in facial view 1.17-1.24x as broad as high; POL 2.0-2.5x OOL, OOL 1.5-2.0x diameter of ocelli; compound eyes with scattered, very short setae (Fig. 2a); lower edge of antennal toruli at the level or even slightly below lower ocular line, 2.2-2.7x closer to clypeal margin than to median ocellus; clypeus moderately emarginated (Fig. 2b); malar space 0.43-0.55x eye height; width of oral fossa 2-2.13x malar space. Scape 0.8-0.9x height of eye, extending far below median ocellus (Fig. 1c); pedicel plus flagellum 0.9x breadth of head; pedicel 1.6-2.0x as long as the first funicular segment; antenna with 3 anelli, first anellus strongly transverse, second and third anellus nearly equal in length, less transverse and about 2x longer than the first (Figs 1c, 2c); funicular segments 1-3 or 1-4 hardly to slightly longer than broad, 4^th^ to 5^th^ subquadrate to quadrate; flagellum subclavate, clava in lateral view 1.6-2.0x as broad as first funicular segment, with narrow strip of micropilosity on the ventral surface of third segment (Fig. 1d); placoid sensilla in one row on each segment (Fig. 2c); funicular segments with scattered, but well visible basiconic capitate pegs (Fig. 2d).

Mesosoma. 1.52-1.58x as long as broad; pronotal collar long medially, about 1/6 to 1/5 (0.17 to 0.2x) length of mesoscutum (Figs 1b, 2e), not carinate anteriorly, with only slightly raised front margin, sloping vertically to dorsal plane of mesoscutum (Fig. 1c), pronotal neck not visible in dorsal view (Fig. 2e); mesoscutum 1.46-1.55x broader than long, 1.17-1.2x as long as scutellum; Propodeum (Fig. 2f) medially less than half (0.35-0.45x) as long as scutellum; median area between plicae 1.80-2.14x broader than long; spiracles ellipsoid in shape, separated by their minor diameter from the hind margin of metanotum; callus setose, with 15-20 setae. Legs rather stout, hind femora 3.4-4.0x as long as broad.

Fore wing. Marginal vein 1.10-1.23x as long as postmarginal vein and 1.60-1.75x as long as stigmal vein (Fig. 1f). Upper surface of costal cell bare; lower surface with one complete row of setae extending to humeral plate and with some setae scattered over distal third; basal vein with complete row of setae, basal cell bare or having at most one seta close to the basal vein; cubital setal line not developed; speculum open below, extending beyond 0.24-0.39x length of marginal vein; marginal setae presented on the entire apical margin; disc rather densely setose.

Gaster. 1.3-1.7x longer than wide, usually 0.72-0.92x shorter than, sometimes as long as head plus mesosoma; tip of hypopygium situated usually about at middle, rarely slightly before or slightly beyond the middle of gaster length; ovipositor sheaths well visible in ventral view, but hardly or not projecting beyond the last tergite.

Male. Unknown.

#### Diagnosis

*Mesopolobusaskewi* sp. nov. belongs to the group of species that have three anelli, long pronotal collar, which is one-sixth to one-fifth the length of mesoscutum and gaster usually slightly shorter than head plus mesosoma. It runs to couplet 36 in Graham’s key (Graham 1969), but clearly differs from *M.longicollis* (Figs 3, 4) by a combination of characters presented in Table 1.

#### Etymology

The first author is pleased to name this species after Dr. Richard Robinson Askew, one of the most prominent experts in the field of entomology, especially in the studies on Chalcidoidea. His works have been an inspiration for me since the beginning of my interest in the family Pteromalidae. In our correspondence Dr. Askew has always demonstrated his responsiveness and natural modesty and that have impressed me many times.

#### Biology

Unknown.

## Checklists

### Other species collected from *P.abies*

#### Asaphes vulgaris

(Walker, 1834)

80B3652A-FF1C-56C6-AE14-00FD25AD85D8

##### Materials

- **Type status:** Other material. **Occurrence:** recordedBy: Ivaylo Todorov leg.; sex: 6 females, 1 male; occurrenceID: E0520583-23DB-5F7D-8EF8-A1DEF2EA28EA; **Location:** country: Bulgaria; locality: Stara Planina: Chiprovska Mts, W Chiprovtsi; verbatimElevation: 1540 m; decimalLatitude: 43.34583; decimalLongitude: 22.81616; **Event:** verbatimEventDate: 18.VII.2019
- **Type status:** Other material. **Occurrence:** recordedBy: Ivaylo Todorov leg.; sex: 8 females, 5 males; occurrenceID: ADB6C85C-DEDC-5A03-9742-A04DBE8E7741; **Location:** country: Bulgaria; locality: Stara Planina: Chiprovska Mts, W Chiprovtsi; verbatimElevation: 1540 m; decimalLatitude: 43.34583; decimalLongitude: 22.81616; **Event:** verbatimEventDate: 17.VII.2020
- **Type status:** Other material. **Occurrence:** recordedBy: Ivaylo Todorov leg.; sex: 2 female; occurrenceID: DD8732A8-C9C8-5D1A-B90D-3DF91DEAB7EE; **Location:** country: Bulgaria; locality: Stara Planina: Shipchenska Mts, near Partizanska pesen hut; verbatimElevation: 1185 m; decimalLatitude: 42.78369; decimalLongitude: 25.19775; **Event:** verbatimEventDate: 26.VII.2019
- **Type status:** Other material. **Occurrence:** recordedBy: Ivaylo Todorov leg.; sex: 152 females, 7 males; occurrenceID: 87E42CD5-6400-56B8-8C33-90EE57FF6DFA; **Location:** country: Bulgaria; locality: Stara Planina: Troyanska Mts, near Beklemeto; verbatimElevation: 1433 m; decimalLatitude: 42.78280; decimalLongitude: 24.62160; **Event:** verbatimEventDate: 17.VIII.2021
- **Type status:** Other material. **Occurrence:** recordedBy: Ivaylo Todorov leg.; sex: 10 females; occurrenceID: 360826DF-D15B-5DD9-A5A7-7A4D8116609C; **Location:** country: Bulgaria; locality: Western Rhodope Mts, near Pamporovo; verbatimElevation: 1580 m; decimalLatitude: 41.62250; decimalLongitude: 24.70100; **Event:** verbatimEventDate: 15.VII.2021

##### Distribution

Probably native to Europe or at most to Western Palearctic and later introduced to North America. Records from Afrotropical and Neotropical Regions, as well as those from Greenland, have most likely been based on misidentifications (Gibson and Vikberg 1998). In Australasian fauna, *A.vulgaris* also has probably been introduced from Europe (Bouček 1988).

#### Mesopolobus dubius

(Walker, 1834)

B429F676-7F74-529A-8CDD-5FB642C8B095

##### Materials

- **Type status:** Other material. **Occurrence:** recordedBy: Ivaylo Todorov leg.; sex: 49 females; occurrenceID: CF590790-4271-5095-BA2F-AAA14D820E6B; **Location:** country: Bulgaria; locality: Stara Planina: Chiprovska Mts, W Chiprovtsi; verbatimElevation: 1540 m; decimalLatitude: 43.34583; decimalLongitude: 22.81616; **Event:** verbatimEventDate: 18.VII.2019
- **Type status:** Other material. **Occurrence:** recordedBy: Ivaylo Todorov leg.; sex: 16 females; occurrenceID: B2E82C8B-E31C-5AF0-9E09-C3DC1957C60A; **Location:** country: Bulgaria; locality: Stara Planina: Shipchenska Mts, near Partizanska pesen hut; verbatimElevation: 1185 m; decimalLatitude: 42.78369; decimalLongitude: 25.19775; **Event:** verbatimEventDate: 26.VII.2019
- **Type status:** Other material. **Occurrence:** recordedBy: Ivaylo Todorov leg.; sex: 2 females, 5 males; occurrenceID: 714CA4B6-147F-58D3-BCFE-C898E07F2016; **Location:** country: Bulgaria; locality: Stara Planina: Shipchenska Mts, near Uzana hut; verbatimElevation: 1243 m; decimalLatitude: 42.75800; decimalLongitude: 25.23427; **Event:** verbatimEventDate: 08.VII.2020
- **Type status:** Other material. **Occurrence:** recordedBy: Ivaylo Todorov leg.; sex: 2 females; occurrenceID: 019DA245-C548-58F9-82F3-A5AB2E5BDE08; **Location:** country: Bulgaria; locality: Stara Planina: Troyanska Mts, near Beklemeto; verbatimElevation: 1433 m; decimalLatitude: 42.78280; decimalLongitude: 24.62160; **Event:** verbatimEventDate: 17.VIII.2021
- **Type status:** Other material. **Occurrence:** recordedBy: Ivaylo Todorov leg.; sex: 1 female; occurrenceID: 43728DFB-8A06-518A-84DC-80573791F31E; **Location:** country: Bulgaria; locality: Western Rhodope Mts, near Pamporovo; verbatimElevation: 1580 m; decimalLatitude: 41.62250; decimalLongitude: 24.70100; **Event:** verbatimEventDate: 24.VII.2019
- **Type status:** Other material. **Occurrence:** recordedBy: Ivaylo Todorov leg.; sex: 1 female; occurrenceID: A787725D-ABA2-5480-A005-8CE45B32B4C5; **Location:** country: Bulgaria; locality: Western Rhodope Mts, near Golyam Perelik peak; verbatimElevation: 2121 m; decimalLatitude: 41.59669; decimalLongitude: 24.58616; **Event:** verbatimEventDate: 15.VIII.2019
- **Type status:** Other material. **Occurrence:** recordedBy: Ivaylo Todorov leg.; sex: 1 female; occurrenceID: 4DCE3E04-FFFE-5E78-8138-7EC7A9B008CB; **Location:** country: Bulgaria; locality: Western Rhodope Mts, S Golyam Perelik peak; verbatimElevation: 2155 m; decimalLatitude: 41.60092; decimalLongitude: 24.57888; **Event:** verbatimEventDate: 15.VIII.2019

##### Distribution

Europe and north-western Turkey. New record for the Bulgarian fauna.

#### Mesopolobus morys

(Walker, 1848)

20E73BCB-869B-540C-8AF5-57D708F79C11

##### Materials

- **Type status:** Other material. **Occurrence:** recordedBy: Ivaylo Todorov leg.; sex: 3 females; occurrenceID: 7C85EC8F-4FEF-52ED-AE45-CB2F13F82329; **Location:** country: Bulgaria; locality: Stara Planina: Chiprovska Mts, W Chiprovtsi; verbatimElevation: 1540 m; decimalLatitude: 43.34583; decimalLongitude: 22.81616; **Event:** verbatimEventDate: 18.VII.2019
- **Type status:** Other material. **Occurrence:** recordedBy: Ivaylo Todorov leg.; sex: 137 females; occurrenceID: AB21F320-747B-5102-B189-179B68E48A9C; **Location:** country: Bulgaria; locality: Stara Planina: Chiprovska Mts, W Chiprovtsi; verbatimElevation: 1540 m; decimalLatitude: 43.34583; decimalLongitude: 22.81616; **Event:** verbatimEventDate: 17.VII.2020
- **Type status:** Other material. **Occurrence:** recordedBy: Ivaylo Todorov leg.; sex: 21 females; occurrenceID: EACC806A-8C5E-536D-BB65-39B5ABCEBA3F; **Location:** country: Bulgaria; locality: Stara Planina: Troyanska Mts, near Beklemeto; verbatimElevation: 1433 m; decimalLatitude: 42.78280; decimalLongitude: 24.62160; **Event:** verbatimEventDate: 17.VIII.2021
- **Type status:** Other material. **Occurrence:** recordedBy: Ivaylo Todorov leg.; sex: 1 female; occurrenceID: DAEE9F4E-B802-599A-A918-8C8CB2E7CDE5; **Location:** country: Bulgaria; locality: Stara Planina: Shipchenska Mts, near Uzana hut; verbatimElevation: 1243 m; decimalLatitude: 42.75800; decimalLongitude: 25.23427; **Event:** verbatimEventDate: 08.VII.2020
- **Type status:** Other material. **Occurrence:** recordedBy: Ivaylo Todorov leg.; sex: 1 female; occurrenceID: 7DC47B8E-8F32-5EB5-A40F-9365B97575E8; **Location:** country: Bulgaria; locality: Western Rhodope Mts, near Pamporovo; verbatimElevation: 1580 m; decimalLatitude: 41.62250; decimalLongitude: 24.70100; **Event:** verbatimEventDate: 24.VII.2019
- **Type status:** Other material. **Occurrence:** recordedBy: Ivaylo Todorov and P. Boyadzhiev leg.; sex: 3 females; occurrenceID: 45507FAE-A9F6-531D-A82F-FADA6905F724; **Location:** country: Bulgaria; locality: Western Rhodope Mts, near Pamporovo; verbatimElevation: 1580 m; decimalLatitude: 41.62250; decimalLongitude: 24.70100; **Event:** verbatimEventDate: 15.VII.2021
- **Type status:** Other material. **Occurrence:** recordedBy: Ivaylo Todorov leg.; sex: 2 females; occurrenceID: 6A38F59C-E253-5775-AD83-20704F0D15F8; **Location:** country: Bulgaria; locality: Western Rhodope Mts, near Golyam Perelik peak; verbatimElevation: 2121 m; decimalLatitude: 41.59669; decimalLongitude: 24.58616; **Event:** verbatimEventDate: 15.VIII.2019
- **Type status:** Other material. **Occurrence:** recordedBy: Ivaylo Todorov leg.; sex: 1 female; occurrenceID: 355B5FD2-F8FD-55F4-ADE2-FDF8B28F475D; **Location:** country: Bulgaria; locality: Western Rhodope Mts, S Golyam Perelik peak; verbatimElevation: 2155 m; decimalLatitude: 41.60092; decimalLongitude: 24.57888; **Event:** verbatimEventDate: 15.VIII.2019

##### Distribution

Western Palearctic and Nearctic.

#### Mesopolobus tibialis

(Westwood, 1833)

13C50D8B-E169-5A55-BD3F-EEDA9DE51E5D

##### Materials

- **Type status:** Other material. **Occurrence:** recordedBy: Ivaylo Todorov leg.; sex: 63 females; occurrenceID: DD42F45A-901B-5700-B7B3-A654CF597FD8; **Location:** country: Bulgaria; locality: Stara Planina: Chiprovska Mts, W Chiprovtsi; verbatimElevation: 1540 m; decimalLatitude: 43.34583; decimalLongitude: 22.81616; **Event:** verbatimEventDate: 18.VII.2019
- **Type status:** Other material. **Occurrence:** recordedBy: Ivaylo Todorov leg.; sex: 31 females; occurrenceID: 9A4FB2EC-45C5-52D4-88D1-9BEB5A1C8AC5; **Location:** country: Bulgaria; locality: Stara Planina: Chiprovska Mts, W Chiprovtsi; verbatimElevation: 1540 m; decimalLatitude: 43.34583; decimalLongitude: 22.81616; **Event:** verbatimEventDate: 17.VII.2020
- **Type status:** Other material. **Occurrence:** recordedBy: Ivaylo Todorov leg.; sex: 2 females; occurrenceID: C9E9D4E3-38BE-59C5-A5F6-128EA84F2AE1; **Location:** country: Bulgaria; locality: Stara Planina: Troyanska Mts, near Beklemeto; verbatimElevation: 1433 m; decimalLatitude: 42.78280; decimalLongitude: 24.62160; **Event:** verbatimEventDate: 17.VIII.2021
- **Type status:** Other material. **Occurrence:** recordedBy: Ivaylo Todorov leg.; sex: 5 females; occurrenceID: 1A722CA2-841A-5DB0-BB04-6ADDC77CC5E5; **Location:** country: Bulgaria; locality: Stara Planina: Berkovska Mts, NW Gintsi vill.; verbatimElevation: 1295 m; decimalLatitude: 43.11366; decimalLongitude: 23.10066; **Event:** verbatimEventDate: 17.VII.2019
- **Type status:** Other material. **Occurrence:** recordedBy: Ivaylo Todorov leg.; sex: 4 females; occurrenceID: 98384EAE-C9E8-52F5-A550-E66A22B58689; **Location:** country: Bulgaria; locality: Stara Planina: Shipchenska Mts, near Partizanska pesen hut; verbatimElevation: 1185 m; decimalLatitude: 42.78369; decimalLongitude: 25.19775; **Event:** verbatimEventDate: 26.VII.2019
- **Type status:** Other material. **Occurrence:** recordedBy: Ivaylo Todorov leg.; sex: 6 females; occurrenceID: 72649AE6-39BE-51AE-84C2-07FD75646AE1; **Location:** country: Bulgaria; locality: Stara Planina: Shipchenska Mts, near Uzana hut; verbatimElevation: 1243 m; decimalLatitude: 42.75800; decimalLongitude: 25.23427; **Event:** verbatimEventDate: 08.VII.2020
- **Type status:** Other material. **Occurrence:** recordedBy: Ivaylo Todorov and P. Boyadzhiev leg.; sex: 12 females; occurrenceID: EEF40899-0F4F-5F50-8F9B-E11A70D04427; **Location:** country: Bulgaria; locality: Western Rhodope Mts, near Pamporovo; verbatimElevation: 1580 m; decimalLatitude: 41.62250; decimalLongitude: 24.70100; **Event:** verbatimEventDate: 24.VII.2019
- **Type status:** Other material. **Occurrence:** recordedBy: Ivaylo Todorov leg.; sex: 12 females; occurrenceID: 1D6B6DC6-46B9-573E-9E6C-4B195B6F4735; **Location:** country: Bulgaria; locality: Western Rhodope Mts, near Perelik hut; verbatimElevation: 2000 m; decimalLatitude: 41.60518; decimalLongitude: 24.59265; **Event:** verbatimEventDate: 20.VIII.2021

##### Distribution

Palearctic.

#### Pachyneuron formosum

Walker, 1833

926BE5DB-56E9-56EB-B17D-0571FB778507

##### Materials

- **Type status:** Other material. **Occurrence:** recordedBy: Ivaylo Todorov leg.; sex: 2 females, 3 males; occurrenceID: 237016CE-DEB0-5A5B-82FB-D87118164F6E; **Location:** country: Bulgaria; locality: Stara Planina: Chiprovska Mts, W Chiprovtsi; verbatimElevation: 1540 m; decimalLatitude: 43.34583; decimalLongitude: 22.81616; **Event:** verbatimEventDate: 17.VII.2020
- **Type status:** Other material. **Occurrence:** recordedBy: Ivaylo Todorov leg.; sex: 153 females, 1 male; occurrenceID: 8A104A9F-2CC4-5AFE-BC33-7D9C2BB8DF6C; **Location:** country: Bulgaria; locality: Stara Planina: Troyanska Mts, near Beklemeto; verbatimElevation: 1433 m; decimalLatitude: 42.78280; decimalLongitude: 24.62160; **Event:** verbatimEventDate: 17.VIII.2021
- **Type status:** Other material. **Occurrence:** recordedBy: Ivaylo Todorov leg.; sex: 75 females; occurrenceID: B1D69C96-DF47-57CB-A11F-CBED7B60A924; **Location:** country: Bulgaria; locality: Western Rhodope Mts, near Perelik hut; verbatimElevation: 2000 m; decimalLatitude: 41.60518; decimalLongitude: 24.59265; **Event:** verbatimEventDate: 20.VIII.2021
- **Type status:** Other material. **Occurrence:** recordedBy: Ivaylo Todorov leg.; sex: 35 females; occurrenceID: 09738CD3-D42A-596A-956C-79B9E447AB50; **Location:** country: Bulgaria; locality: Western Rhodope Mts, N Golyam Perelik peak; verbatimElevation: 2144 m; decimalLatitude: 41.60968; decimalLongitude: 24.57656; **Event:** verbatimEventDate: 20.VIII.2021
- **Type status:** Other material. **Occurrence:** recordedBy: Ivaylo Todorov and P. Boyadzhiev leg.; sex: 42 females, 1 male; occurrenceID: 8B063C2E-81F6-50D1-8D46-3417F2BF7FA2; **Location:** country: Bulgaria; locality: Western Rhodope Mts, near Pamporovo; verbatimElevation: 1580 m; decimalLatitude: 41.62250; decimalLongitude: 24.70100; **Event:** verbatimEventDate: 15.VII.2021

##### Distribution

Palearctic.

#### Stenomalina micans

(Olivier, 1813)

8B3F47A8-8C39-50E0-B5FB-7A00B9D46204

##### Materials

- **Type status:** Other material. **Occurrence:** recordedBy: Ivaylo Todorov leg.; sex: 3 females; occurrenceID: BE77C723-25BA-5CCE-AE7F-F2AA63298441; **Location:** country: Bulgaria; locality: Western Rhodope Mts, near Golyam Perelik peak; verbatimElevation: 2121 m; decimalLatitude: 41.59669; decimalLongitude: 24.58616; **Event:** verbatimEventDate: 15.VIII.2019
- **Type status:** Other material. **Occurrence:** recordedBy: Ivaylo Todorov leg.; sex: 12 females; occurrenceID: 17AC80C3-0753-5D08-9CFA-3E978CF1172F; **Location:** country: Bulgaria; locality: Western Rhodope Mts, S Golyam Perelik peak; verbatimElevation: 2155 m; decimalLatitude: 41.60092; decimalLongitude: 24.57888; **Event:** verbatimEventDate: 15.VIII.2019

##### Distribution

Europe, Kazakhstan, China. New record for the Bulgarian fauna.

#### Trichomalus bracteatus

(Walker, 1835)

A4A39178-73E7-5CCE-9D2A-2D1616B2B7E7

##### Materials

- **Type status:** Other material. **Occurrence:** recordedBy: Ivaylo Todorov leg.; sex: 13 females, 3 males; occurrenceID: 5C5AE605-CE8E-5E87-82BC-4FF8B0DD537F; **Location:** country: Bulgaria; locality: Stara Planina: Chiprovska Mts, W Chiprovtsi; verbatimElevation: 1540 m; decimalLatitude: 43.34583; decimalLongitude: 22.81616; **Event:** verbatimEventDate: 18.VII.2019
- **Type status:** Other material. **Occurrence:** recordedBy: Ivaylo Todorov leg.; sex: 2 females, 6 males; occurrenceID: AFEE316F-786C-5BE2-899E-B892184D08BF; **Location:** country: Bulgaria; locality: Stara Planina: Chiprovska Mts, W Chiprovtsi; verbatimElevation: 1540 m; decimalLatitude: 43.34583; decimalLongitude: 22.81616; **Event:** verbatimEventDate: 17.VII.2020
- **Type status:** Other material. **Occurrence:** recordedBy: Ivaylo Todorov leg.; sex: 4 females; occurrenceID: 0D779A65-DED9-522A-87A7-8C806AD7671F; **Location:** country: Bulgaria; locality: Stara Planina: Shipchenska Mts, near Partizanska pesen hut; verbatimElevation: 1185 m; decimalLatitude: 42.78369; decimalLongitude: 25.19775; **Event:** verbatimEventDate: 26.VII.2019
- **Type status:** Other material. **Occurrence:** recordedBy: Ivaylo Todorov leg.; sex: 1 female; occurrenceID: 13268E2B-1931-583D-9557-9932A06840A9; **Location:** country: Bulgaria; locality: Stara Planina: Shipchenska Mts, near Uzana hut; verbatimElevation: 1243 m; decimalLatitude: 42.75800; decimalLongitude: 25.23427; **Event:** verbatimEventDate: 08.VII.2020
- **Type status:** Other material. **Occurrence:** recordedBy: Ivaylo Todorov leg.; sex: 1 female; occurrenceID: DF29D627-6DBB-52CE-9B92-BB546450D114; **Location:** country: Bulgaria; locality: Western Rhodope Mts, near Pamporovo; verbatimElevation: 1580 m; decimalLatitude: 41.62250; decimalLongitude: 24.70100; **Event:** verbatimEventDate: 15.VII.2021

##### Distribution

Europe. New record for the Bulgarian fauna.

#### Trichomalus helvipes

(Walker, 1834)

78FFC350-EF50-5849-BB73-4C8075083FD1

##### Materials

- **Type status:** Other material. **Occurrence:** recordedBy: Ivaylo Todorov leg.; sex: 41 females, 16 males; occurrenceID: 6A2547FA-956F-5EA8-8DF5-03F4C2297F12; **Location:** country: Bulgaria; locality: Stara Planina: Chiprovska Mts, W Chiprovtsi; verbatimElevation: 1540 m; decimalLatitude: 43.34583; decimalLongitude: 22.81616; **Event:** verbatimEventDate: 18.VII.2019
- **Type status:** Other material. **Occurrence:** recordedBy: Ivaylo Todorov leg.; sex: 13 females, 1 male; occurrenceID: AAF34C42-AC42-5016-B97D-16B2BB278140; **Location:** country: Bulgaria; locality: Stara Planina: Chiprovska Mts, W Chiprovtsi; verbatimElevation: 1540 m; decimalLatitude: 43.34583; decimalLongitude: 22.81616; **Event:** verbatimEventDate: 17.VII.2020
- **Type status:** Other material. **Occurrence:** recordedBy: Ivaylo Todorov leg.; sex: 1 female; occurrenceID: 22E134E3-C9A3-5520-927A-46791781F39C; **Location:** country: Bulgaria; locality: Stara Planina: Shipchenska Mts, near Partizanska pesen hut; verbatimElevation: 1185 m; decimalLatitude: 42.78369; decimalLongitude: 25.19775; **Event:** verbatimEventDate: 26.VII.2019
- **Type status:** Other material. **Occurrence:** recordedBy: Ivaylo Todorov leg.; sex: 19 females, 1 male; occurrenceID: 7B16FACE-6922-5B4D-939C-13A77227D9BE; **Location:** country: Bulgaria; locality: Stara Planina: Troyanska Mts, near Beklemeto; verbatimElevation: 1433 m; decimalLatitude: 42.78280; decimalLongitude: 24.62160; **Event:** verbatimEventDate: 17.VIII.2021
- **Type status:** Other material. **Occurrence:** recordedBy: Ivaylo Todorov leg.; sex: 1 female; occurrenceID: A76BAF3A-15CD-5A1D-9C81-D2FA312D1080; **Location:** country: Bulgaria; locality: Stara Planina: Berkovska Mts, near Kom hut; verbatimElevation: 1248 m; decimalLatitude: 43.19966; decimalLongitude: 23.07550; **Event:** verbatimEventDate: 10.VI.2021

##### Distribution

Europe, Anatolia.

#### Trichomalus inscitus

(Walker, 1835)

FF5E3616-F101-593E-98B8-EAEE4ADD8952

##### Materials

- **Type status:** Other material. **Occurrence:** recordedBy: Ivaylo Todorov leg.; sex: 4 females; occurrenceID: F9E28658-120E-58E0-A044-494ACCFB0D7A; **Location:** country: Bulgaria; locality: Stara Planina: Chiprovska Mts, W Chiprovtsi; verbatimElevation: 1540 m; decimalLatitude: 43.34583; decimalLongitude: 22.81616; **Event:** verbatimEventDate: 18.VII.2019
- **Type status:** Other material. **Occurrence:** recordedBy: Ivaylo Todorov leg.; sex: 19 females, 8 males; occurrenceID: 84820BED-90B2-50FE-AF57-D8B512A06FBD; **Location:** country: Bulgaria; locality: Stara Planina: Chiprovska Mts, W Chiprovtsi; verbatimElevation: 1540 m; decimalLatitude: 43.34583; decimalLongitude: 22.81616; **Event:** verbatimEventDate: 17.VII.2020
- **Type status:** Other material. **Occurrence:** recordedBy: Ivaylo Todorov leg.; sex: 1 female; occurrenceID: 404226CC-A949-579B-A4B9-C9590413C3A7; **Location:** country: Bulgaria; locality: Stara Planina: Chiprovska Mts, W Chiprovtsi; verbatimElevation: 1540 m; decimalLatitude: 43.34583; decimalLongitude: 22.81616; **Event:** verbatimEventDate: 15.VII.2021
- **Type status:** Other material. **Occurrence:** recordedBy: Ivaylo Todorov leg.; sex: 22 females; occurrenceID: 796D7B6A-6C4E-572D-B5C2-411A1F264ADB; **Location:** country: Bulgaria; locality: Stara Planina: Troyanska Mts, near Beklemeto; verbatimElevation: 1433 m; decimalLatitude: 42.78280; decimalLongitude: 24.62160; **Event:** verbatimEventDate: 17.VIII.2021

##### Distribution

Europe, USA.

#### Trichomalus perfectus

(Walker, 1835)

3902A6B6-1666-5886-B455-689A501D7335

##### Materials

- **Type status:** Other material. **Occurrence:** recordedBy: Ivaylo Todorov leg.; sex: 40 females, 59 males; occurrenceID: DD3AA7B7-450C-5EE4-909F-C27F369226F6; **Location:** country: Bulgaria; locality: Stara Planina: Chiprovska Mts, W Chiprovtsi; verbatimElevation: 1540 m; decimalLatitude: 43.34583; decimalLongitude: 22.81616; **Event:** verbatimEventDate: 18.VII.2019
- **Type status:** Other material. **Occurrence:** recordedBy: Ivaylo Todorov leg.; sex: 20 females, 32 males; occurrenceID: EE18FFAB-9BBD-5DBD-AF21-3460F94F102E; **Location:** country: Bulgaria; locality: Stara Planina: Chiprovska Mts, W Chiprovtsi; verbatimElevation: 1540 m; decimalLatitude: 43.34583; decimalLongitude: 22.81616; **Event:** verbatimEventDate: 17.VII.2020
- **Type status:** Other material. **Occurrence:** recordedBy: Ivaylo Todorov leg.; sex: 13 females, 2 males; occurrenceID: D462B896-5D35-56AD-A962-53452A8F0C2A; **Location:** country: Bulgaria; locality: Stara Planina: Shipchenska Mts, near Partizanska pesen hut; verbatimElevation: 1185 m; decimalLatitude: 42.78369; decimalLongitude: 25.19775; **Event:** verbatimEventDate: 26.VII.2019
- **Type status:** Other material. **Occurrence:** recordedBy: Ivaylo Todorov leg.; sex: 1 male; occurrenceID: A97428ED-3090-51B5-87C1-E265B00C3554; **Location:** country: Bulgaria; locality: Stara Planina: Zlatishko-Tetevenska Mts, S Yamna vill.; verbatimElevation: 1014 m; decimalLatitude: 42.81300; decimalLongitude: 24.00233; **Event:** verbatimEventDate: 26.VII.2019
- **Type status:** Other material. **Occurrence:** recordedBy: Ivaylo Todorov leg.; sex: 3 females, 7 males; occurrenceID: 57DFB519-88FA-5810-B251-0162F170CF04; **Location:** country: Bulgaria; locality: Stara Planina: Shipchenska Mts, near Uzana hut; verbatimElevation: 1243 m; decimalLatitude: 42.75800; decimalLongitude: 25.23427; **Event:** verbatimEventDate: 08.VII.2020
- **Type status:** Other material. **Occurrence:** recordedBy: Ivaylo Todorov leg.; sex: 12 females; occurrenceID: AC6E84C2-B740-5A50-8107-29F9206C4E91; **Location:** country: Bulgaria; locality: Stara Planina: Troyanska Mts, near Beklemeto; verbatimElevation: 1433 m; decimalLatitude: 42.78280; decimalLongitude: 24.62160; **Event:** verbatimEventDate: 17.VIII.2021
- **Type status:** Other material. **Occurrence:** recordedBy: Ivaylo Todorov leg.; sex: 1 female; occurrenceID: CA89EACD-A96C-5074-BEFA-5B119FF70C80; **Location:** country: Bulgaria; locality: Western Rhodope Mts, near Pamporovo; verbatimElevation: 1580 m; decimalLatitude: 41.62250; decimalLongitude: 24.70100; **Event:** verbatimEventDate: 15.VII.2021
- **Type status:** Other material. **Occurrence:** recordedBy: Ivaylo Todorov leg.; sex: 1 female; occurrenceID: 19C0AA1C-C693-55FC-8826-0B970169A9E0; **Location:** country: Bulgaria; locality: Western Rhodope Mts, W Orechovo vill., Kostin kamak area; verbatimElevation: 1254 m; decimalLatitude: 41.85500; decimalLongitude: 24.61550; **Event:** verbatimEventDate: 21.VIII.2021

##### Distribution

Western Palearctic and Nearctic Regions. Probably with Holarctic range, but still not reported from North Africa and north-eastern Asia.

## Analysis

### Comparison between M.askewi sp. nov. and some species described after Graham’s monograph (Graham 1969)

Following their original descriptions, females of most species in the genus *Mesopolobus* described after Graham’s monograph, can be easily recognised and distinguished from the pair *longicollis* - *askewi* by a lot of features which are just partly discussed here. Although a few species share some morphological characters with *M.askewi* sp. nov., especially in having antennae with three anelli and pronotum with long collar, they can be well differentiated from this species and are probably not closely allied to it. *Mesopolobusszelenyii* Bouček, 1974 have three anelli and the pronotal collar is a little more than one-fifth the length of mesoscutum (0.18x), but the collar is carinate anteriorly in its middle part, the head is 1.25-1.28x as broad as mesoscutum (1.11-1.16x in *askewi*) and the fore wings have fuscous cross-fascia attached to darker parastigma (hyaline in *askewi*). *Mesopolobusblascoi* Askew, 1994, which was later synonymised under *M.maculipennis* (Mercet) by Garrido and Nieves Aldrey (1996), has a very long collar - one-third to one-fifth the length of mesoscutum, the antennal flagellum has its second anellus subquadrate and the third one is longer than broad, about as long as the first two. Moreover, the venation of the fore wings in *maculipennis* is very characteristic – the postmarginal vein is about as long as the stigmal vein. Another species described by Askew, *M.semenis* Askew, 1997, has a collar 0.19x (1:5.2) as long as the mesoscutum, but differs from *M.askewi* sp. nov. in having the clypeus with a median incision flanked by two blunt teeth, the scape as long as the height of eye and the basal vein bare. Another species with a relatively long pronotal collar (0.15x length of mesoscutum), *M.fagi* Askew and Lampe, 1998, may be distinguished from *M.askewi* sp. nov. by its more clavate flagellum, almost quadrate third anellus and basal vein without setae. The Maltese species *M.melitensis* Askew, 2014, whose collar is almost one-quarter the length of the mesoscutum, has its general colouration distinctly different compared to *M.askewi* sp. nov. - head and mesosoma in *melitensis* are purplish-black, the pedicel is light brown, the funicular segments, except the fifth are darker brown and the clava is pale yellow. Very close to *M.longicollis* is the Chinese species *M.mesolatus* Sun, Xiao and Xu, 2005, which can be distinguished by the characters presented in Sun et al. (2005). This species partly resembles *M.askewi* sp. nov. in the colouration of the mesosoma, but has the antennal toruli a little above the ventral edge of the eyes, the postmarginal vein nearly as long as the stigmal vein, the disc of fore wing with sparse setation and the speculum extending beneath the marginal vein to the stigmal vein as a broad, bare strip. The last species whose female resembles the female of *M.askewi* sp. nov. in some aspects is *M.robiniae* Lakatos and László, 2021. This species has a moderately long collar, one-fifth to one-sixth the length of mesoscutum, but could be easily differentiated from *askewi* sp. nov. through its longer scape that almost reaches the lower edge of the median ocellus, the basal vein bare or with one to three setae and in having the marginal vein 2.00–2.47x longer than the stigmal vein.

### Molecular results

Two mitochondrial sequences were obtained in total. The COI amplicon (663 bp) was successfully sequenced using only the forward primer, while the CYTB amplicon (756 bp) was sequenced with both the forward and reverse primers. The COI sequence showed a difference of 45 base pairs compared to the *Mesopolobusamaenus* (Walker, 1834) sequence recorded in GenBank, while the CYTB sequence differed by 91 base pairs from *Pteromaluspuparum* (Linnaeus, 1758). Both unique sequences were uploaded to the GenBank database under accession numbers PQ331194 for COI and PQ342001 for CYTB.

## Discussion

Тhe species collected during the present study belong mostly to the family Pteromalidae, with the exception of *A.vulgaris*, which was recently classified as Asaphesinae *incertae sedis* in Chalcidoidea (Burks et al. 2022).

The collected species have various host preferences within different insect groups. They are mostly larval ectoparasitoids and only *S.micans* has been proposed as a probable larval endoparasitoid of the Barley Gout-fly, *Chloropstaeniopus* Meigen (Graham and Claridge 1965). Two species, *A.vulgaris* and *P.formosum*, are well known polyphagous hyperparasitoids on aphids (Hemiptera, Aphididae) through their apidiine (Hymenoptera, Braconidae, Aphidiinae) or Ichneumonidae (Hymenoptera) primary parasitoid hosts, respectively. However, *A.vulgaris* was also reported to attack many hosts in two other orders – Coleoptera and Diptera (Noyes 2019, UCD Community 2023).

The three *Mesopolobus* species have different host associations, but a certain similarity is known for *M.dubius* and *M.tibialis*, which are parasitoids in galls of Cynipidae (Hymenoptera, Cynipoidea) on various *Quercus* species. However, two records of *M.dubius* associated with the invasive gall-wasp *Dryocosmuskuriphilus* Yasumatsu (Hymenoptera, Cynipidae) on the sweet chestnut *Castaneasativa* Mill. have also been published in the past (Matošević and Melika 2013). On other hand, *M.morys* attacks mostly larvae of *Ceutorhynchus* spp. (Coleoptera, Curculionidae) living on Brassicaceae (Noyes 2019, UCD Community 2023).

The host of the four *Trichomalus* species are quite different, both in biological and ecological aspects. Three of them, *T.bracteatus*, *T.helvipes* and *T.perfectus*, prefer to parasitise stem-boring larvae living in grasses, while *T.inscitus* is an oligophagous ectoparasitoid mostly of the flea weevils belonging to genus *Orchestes* and rarely of other curculionids such as *Rhamphus*, *Rhynchaenus* and *Stereonychus* associated with various tree hosts (Noyes 2019, UCD Community 2023, Todorov et al. 2024). In addition, *T.bracteatus* was reported to emerge from galls of the cynipid wasp *Biorhizapallida* (Olivier) Thompson (1958) and *T.perfectus* is known as a key natural enemy of the cabbage seed weevil, *Ceutorhynchusobstrictus* (Ulber et al. 2010), a major pest of some Brassicaceae crops.

Considering published data on the biology of the species discussed above, it is surprising to find them associated with *P.abies*. This statement is definitely clear for species like *M.dubius* and *M.tibialis*, whose trophic relationships mostly include hosts on oaks, for *M.morys* which attacks weevils’ larvae in Brassicaceae grass plants and for all *Trichomalus* spp. having in mind their known hosts living on grasses or deciduous trees. A possible association with conifers has been recorded only for *A.vulgaris* and *M.tibialis* with *Pinushalepensis* Mill. (Askew et al. 2001) and for *S.micans* with two bark beetles, *Pityogenesbistridentatus* (Eichhoff) and *Polygraphuspoligraphus* (L.) (Coleoptera, Scolytidae) (Herting 1973).

In this study, a great number of jumping plant-lice belonging to the psyllid genus *Cacopsylla* (Hemiptera, Psyllidae) were collected from the lower branches of the Norway spruce in most of the sampling sites. These insects, as well as all Psyllidae, are well known honeydew producers. Their immature to adult life cycle is restricted to one or a few closely-related host-plant species (Burckhardt et al. 2014) and predominantly completes on perennial dicotyledonous angiosperms (Ripka et al. 2018). However, many psyllids can be observed on conifers where they overwinter to find shelter (Bodnár et al. 2022).

Therefore, acknowledging the biology of the discussed chalcidoids and the attraction of many Pteromalidae to honeydew produced by hemipterans (aphids) (Bouček and Rasplus 1991), we hypothesise that the great overall number of the collected chalcidoid specimens in the present study is not the result of their host associations, but rather represents an aggregation caused by their attraction towards an available food source of the psyllid’s honeydew.

## Supplementary Material

XML Treatment for Mesopolobus askewi

XML Treatment for Asaphes vulgaris

XML Treatment for Mesopolobus dubius

XML Treatment for Mesopolobus morys

XML Treatment for Mesopolobus tibialis

XML Treatment for Pachyneuron formosum

XML Treatment for Stenomalina micans

XML Treatment for Trichomalus bracteatus

XML Treatment for Trichomalus helvipes

XML Treatment for Trichomalus inscitus

XML Treatment for Trichomalus perfectus

## Figures and Tables

**Figure 1a.**
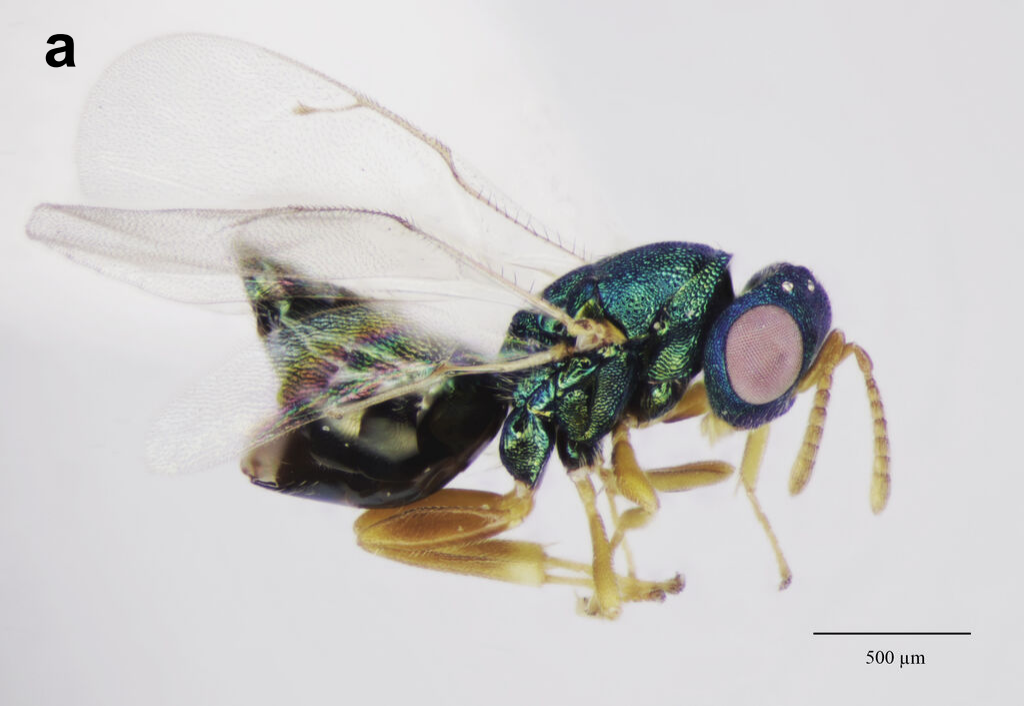
Holotype habitus, lateral view;

**Figure 1b.**
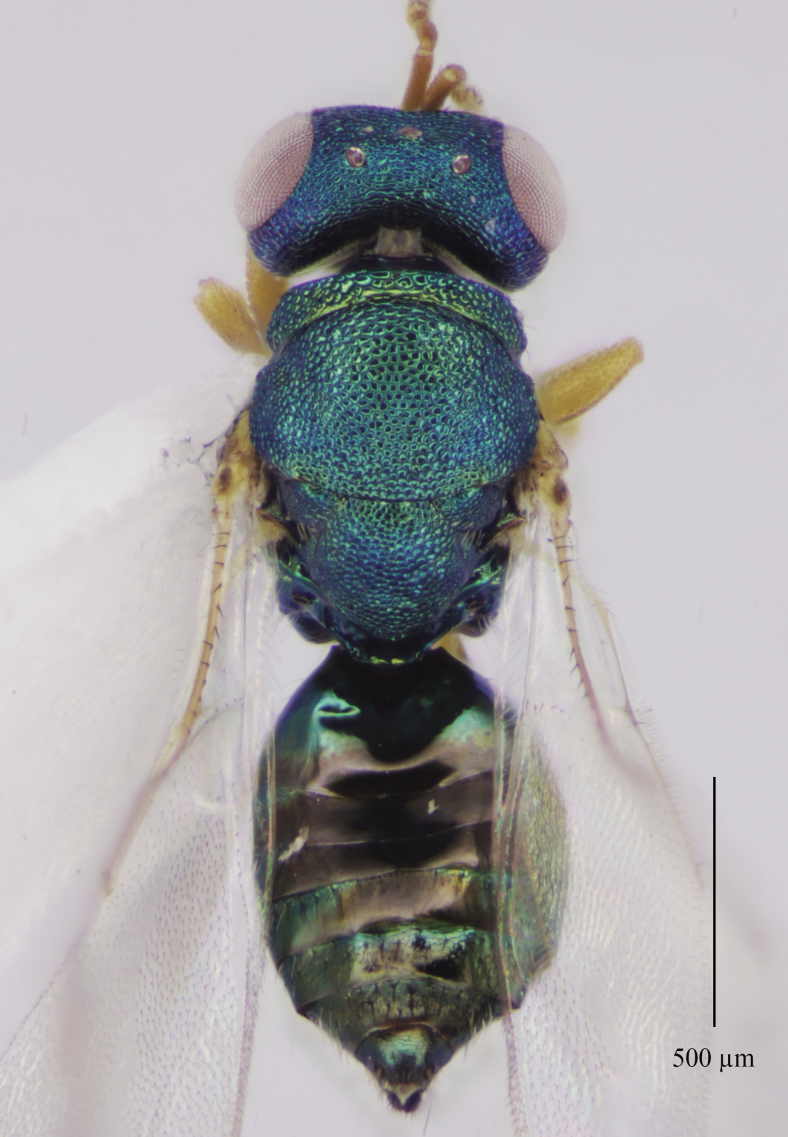
Holotype, dorsal view;

**Figure 1c.**
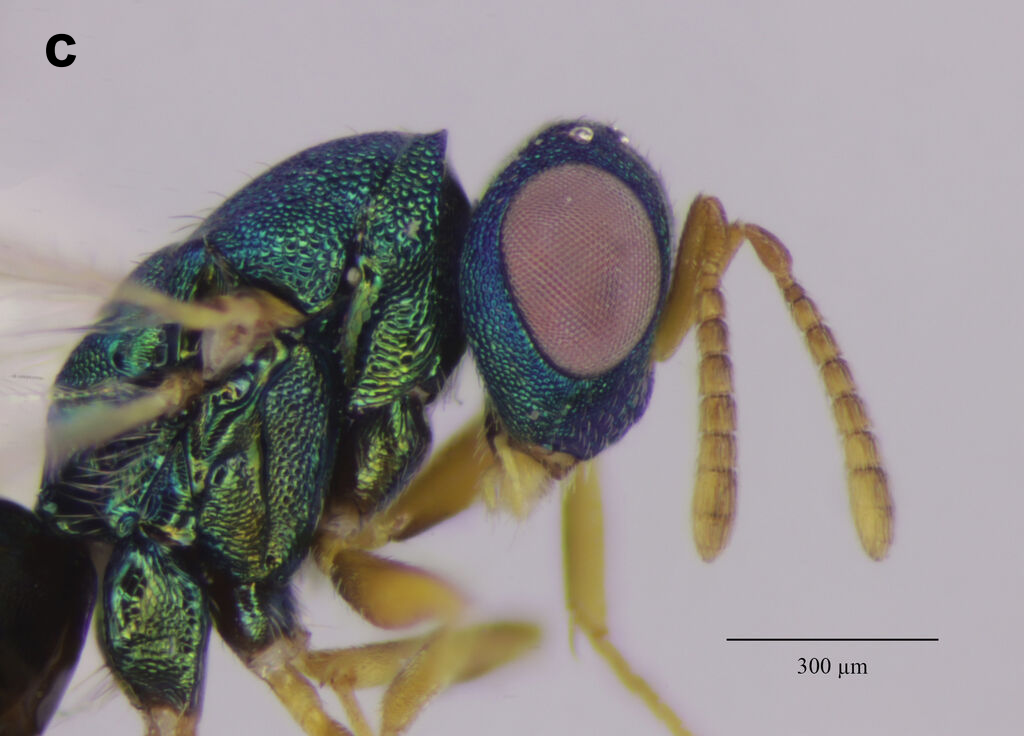
Holotype head and mesosoma, lateral view;

**Figure 1d.**
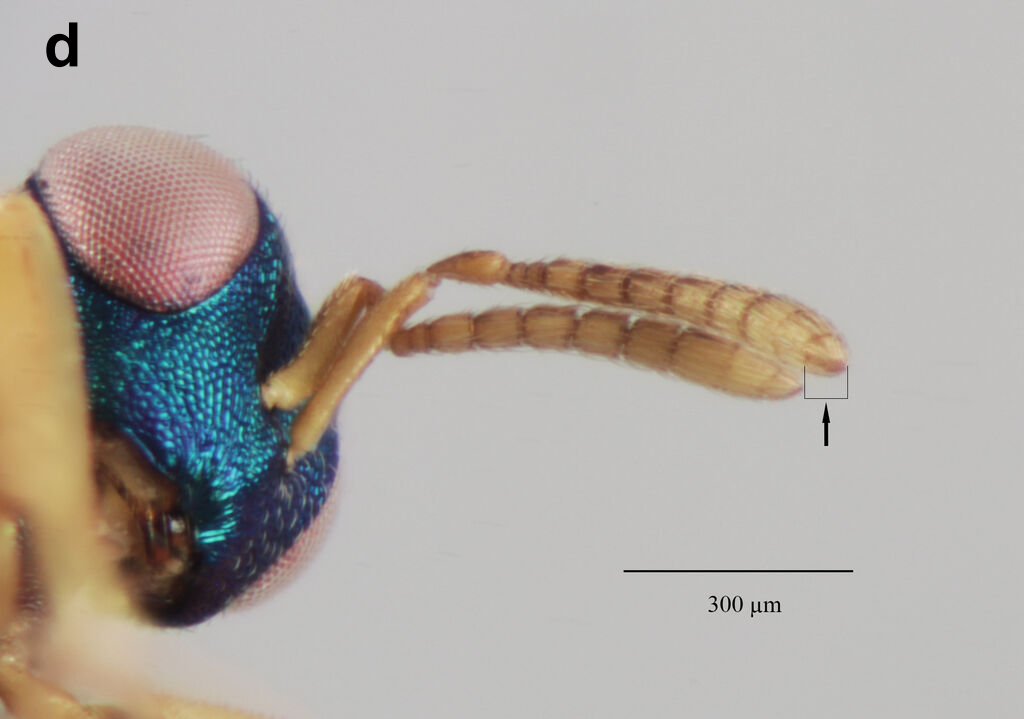
Paratype claval strip of micropilosity (indicated with black lines and an arrow);

**Figure 1e.**
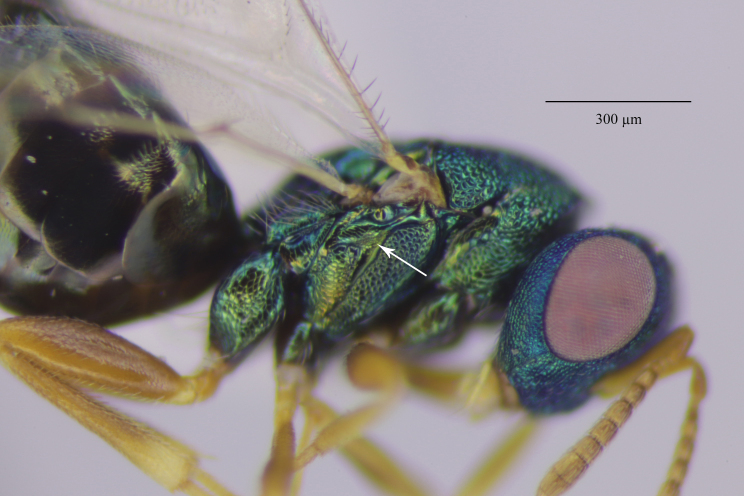
Holotype upper mesepimeron (pointed with an arrow);

**Figure 1f.**
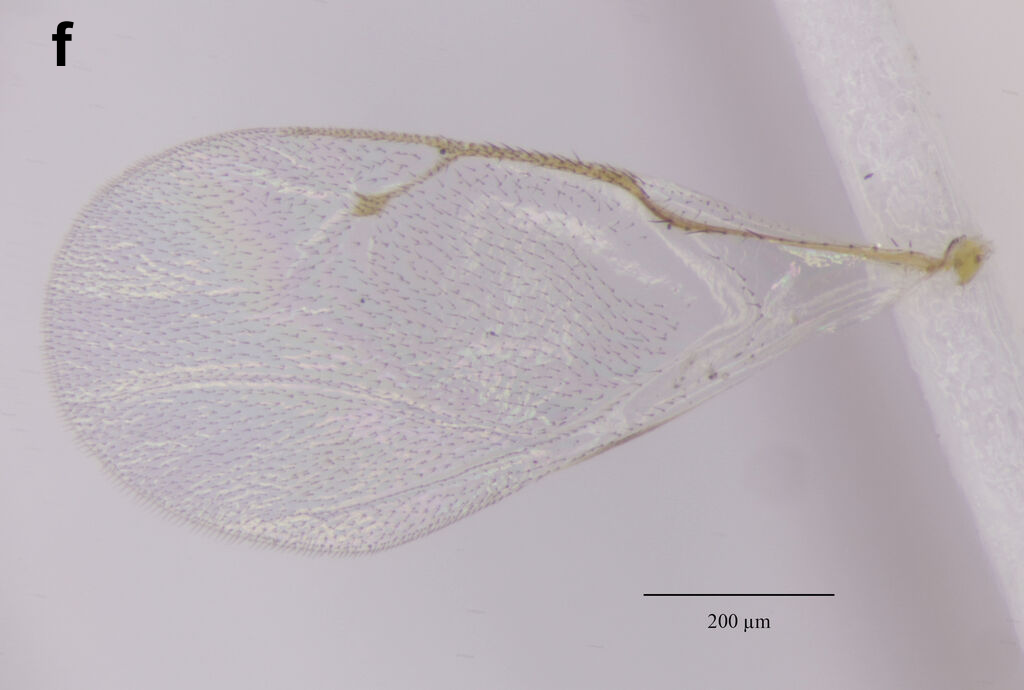
Paratype fore wing.

**Figure 2a.**
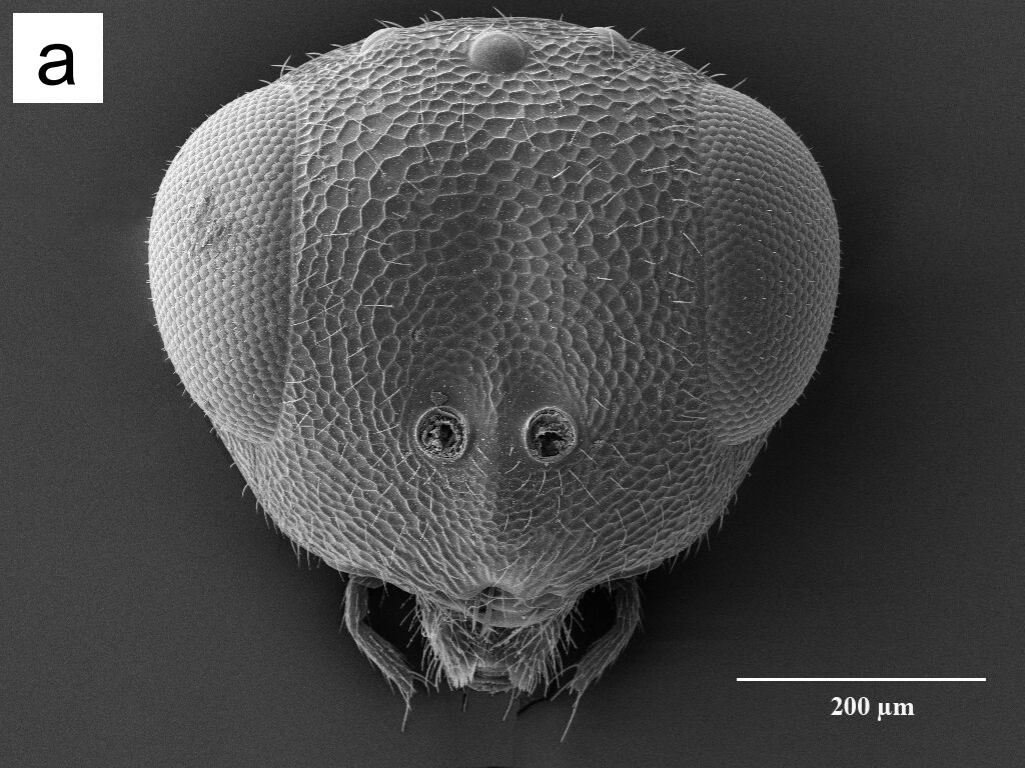
Head, facial view;

**Figure 2b.**
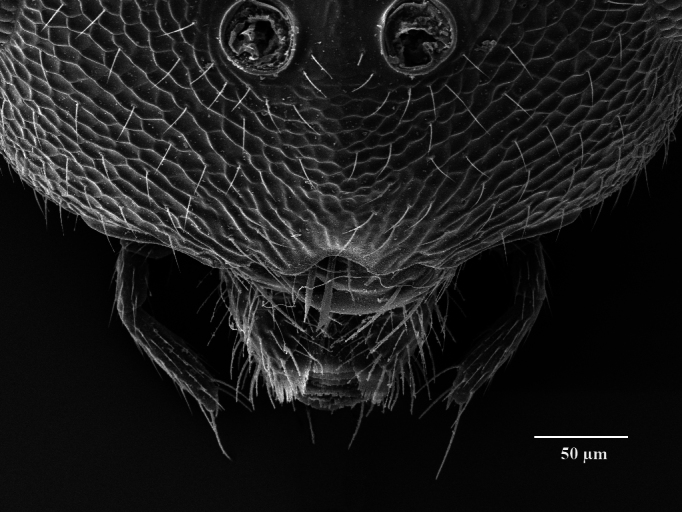
Clypeus;

**Figure 2c.**
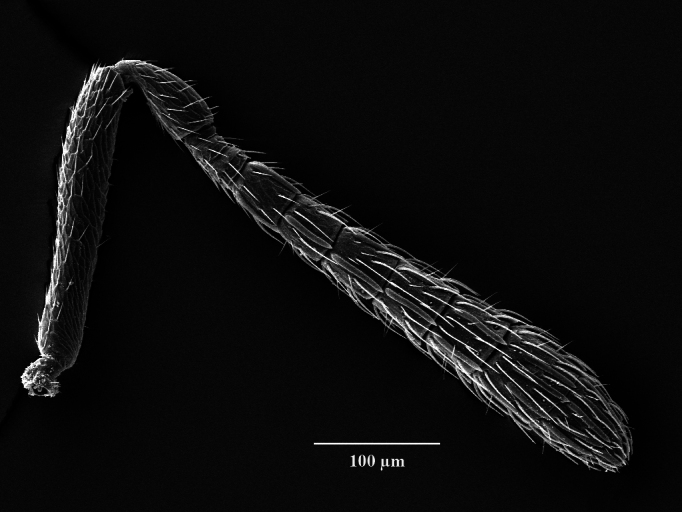
Antenna, lateral view;

**Figure 2d.**
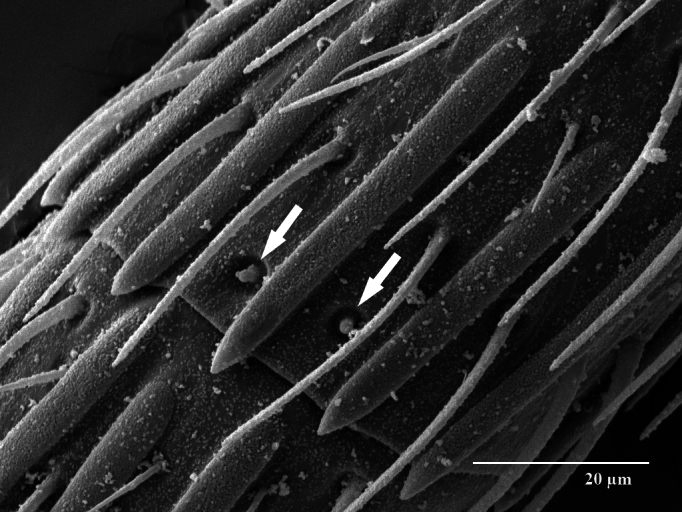
Antennal basiconic capitate pegs (pointed with white arrows);

**Figure 2e.**
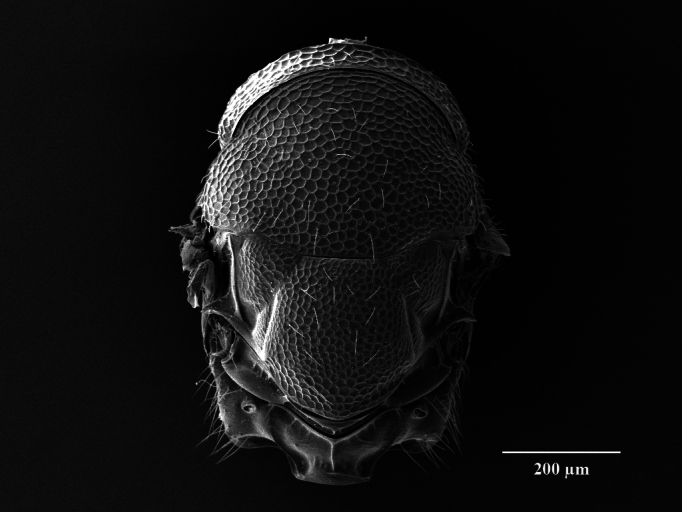
Mesosoma, dorsal view;

**Figure 2f.**
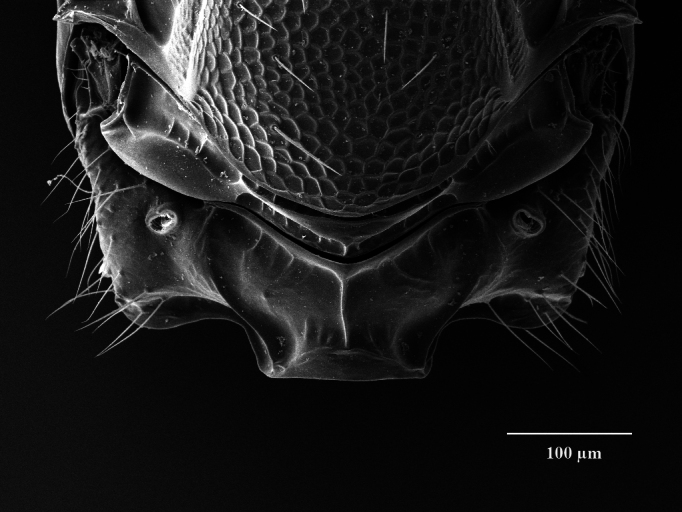
Propodeum.

**Figure 3a.**
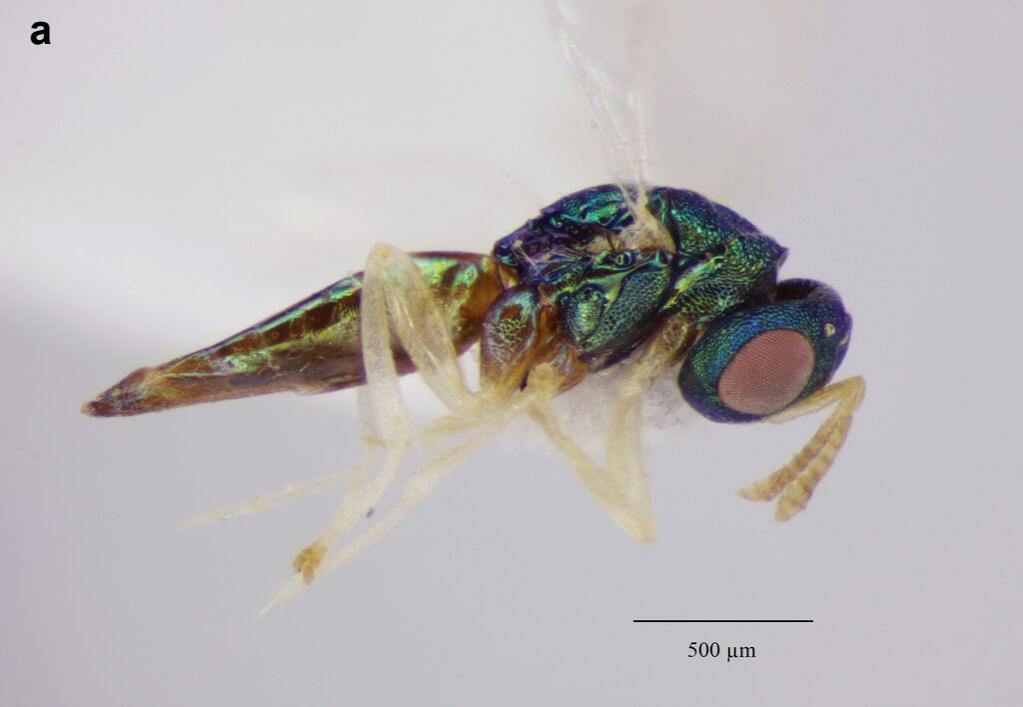
Habitus, lateral view;

**Figure 3b.**
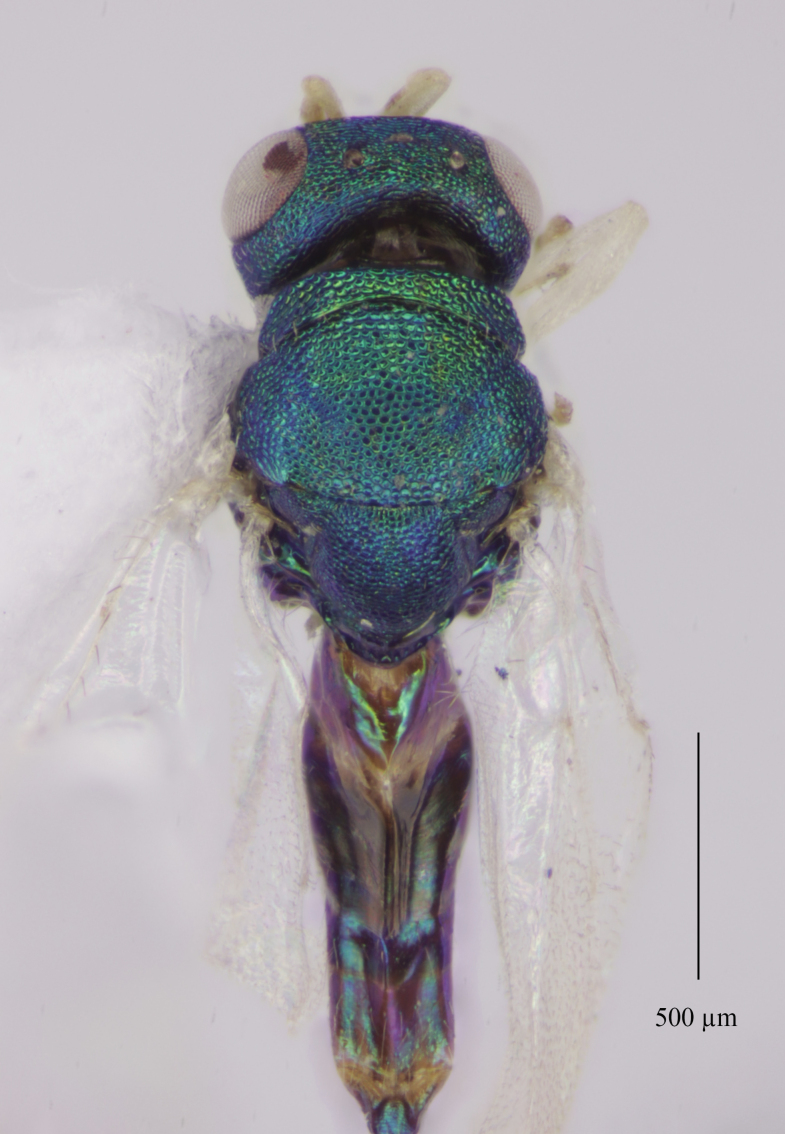
Dorsal view.

**Figure 4a.**
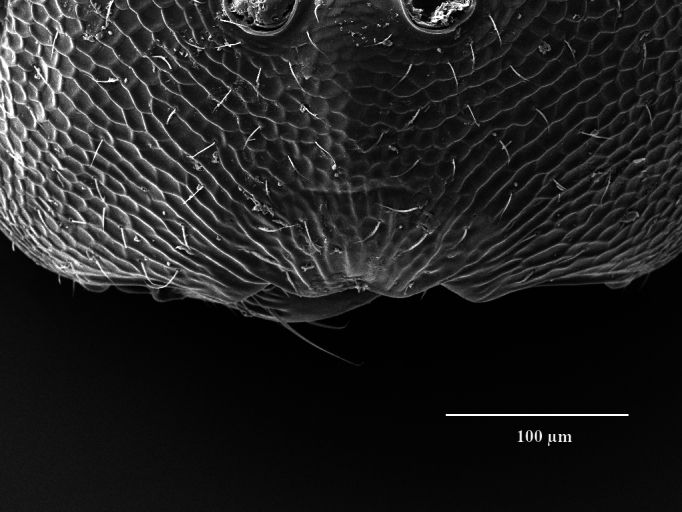
Female clypeus;

**Figure 4b.**
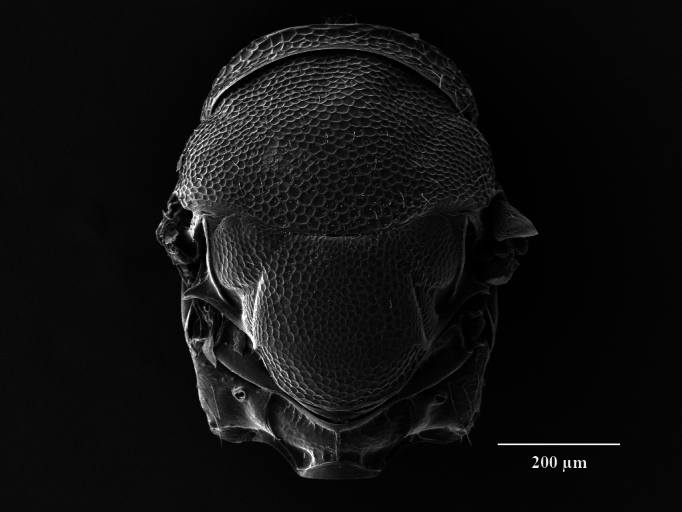
Female mesosoma, dorsal view.

**Table 1.** Updated key to females of *M.askewi* sp. nov. and *M.longicollis* (characters in Bold are interpolated in the couplets designed by Graham (1969) to improve the key). Considering the original terms, we use thorax instead of mesosoma, but understand them as being equivalent.

36 (34)	Pronotal collar (Text-fig. 532) long, medially one-seventh to **one-fifth** as long as the mesoscutum, coarsely reticulate. Squat species with gaster **usually** slightly shorter than, rarely as long as, **the combined length of** head plus thorax, at least slightly less than twice as long as broad. **Thorax golden green or bluish-green, head golden green to blue**	37
-	Either the pronotal collar, medially, is at most one-eighth as long as the mesoscutum; or the gaster is about as long as head plus thorax and at least twice as long as broad and the head and thorax are bronze-green to bronze	38
37 (36)	Anterior margin of clypeus shallowly emarginated (Fig. 4a); POL 1.9-**2.4x** OOL; pronotal collar medially about 1/7 to 1/6 (0.14 to 0.17)x length of mesoscutum (Figs 3b, 4b); fore wing with basal vein bare or with 1-2 setae, basal cell bare; head and thorax green to bluish-green, sometimes with golden reflections; legs beyond coxae pale yellow (Fig. 3a); protarsi with fifth segment fuscous; venation pale yellow	*M.longicollis*
-	Anterior margin of clypeus moderately emarginated (Fig. 2b); POL 2.0-2.5x OOL; pronotal collar sometimes longer, medially about 1/6 to 1/5 (0.17 to 0.2)x length of mesoscutum (Figs 1b, 2e); fore wing having basal vein with complete row of setae, basal cell bare or sometimes with one seta close to the basal vein (Fig. 1f); head blue to bluish-green, thorax bluish-green to green with coppery reflections; legs beyond coxae mostly fulvous, only distal one-fifth of tibiae yellowish; protarsi with fifth segment yellowish, only tarsal claws fuscous; venation pale testaceous	*M.askewi* sp. nov.
38 (36)	= 37 (36) in the key of Graham (1969) etc.	
